# Concomitant occurrence of *EGFR (epidermal growth factor receptor)* and *KRAS (V-Ki-ras2 Kirsten rat sarcoma viral oncogene homolog)* mutations in an *ALK (anaplastic lymphoma kinase)*-positive lung adenocarcinoma patient with acquired resistance to crizotinib: a case report

**DOI:** 10.1186/1756-0500-6-489

**Published:** 2013-11-26

**Authors:** Henrik H Rossing, Morten Grauslund, Edyta M Urbanska, Linea C Melchior, Charlotte K Rask, Junia C Costa, Birgit G Skov, Jens Benn Sørensen, Eric Santoni-Rugiu

**Affiliations:** 1 Laboratory of Molecular Pathology, Department of Pathology, Rigshospitalet, Copenhagen University Hospital, 9 Blegdamsvej, 2100 Copenhagen, Denmark; 2 Department of Oncology, Rigshospitalet, Copenhagen University Hospital, 9 Blegdamsvej, 2100 Copenhagen, Denmark; 3 Department of Clinical Physiology, Nuclear Medicine and PET, Rigshospitalet, Copenhagen University Hospital, 9 Blegdamsvej, 2100 Copenhagen, Denmark; 4 Department of Radiotherapy, Rigshospitalet, Copenhagen University Hospital, 9 Blegdamsvej, 2100 Copenhagen, Denmark; 5 Department of Pathology, Bispebjerg Section, Rigshospitalet, Copenhagen University Hospital, 23 Bispebjerg Bakke, 2400 Copenhagen, Denmark

**Keywords:** Lung cancer, *EML4-ALK*, Crizotinib, Acquired resistance, *EGFR* mutation, *KRAS* mutation

## Abstract

**Background:**

*Anaplastic lymphoma kinase*-positive non-small cell lung carcinoma patients are generally highly responsive to the dual anaplastic lymphoma kinase and MET tyrosine kinase inhibitor crizotinib. However, they eventually acquire resistance to this drug, preventing the anaplastic lymphoma kinase inhibitors from having a prolonged beneficial effect. The molecular mechanisms responsible for crizotinib resistance are beginning to emerge, e.g., in some *anaplastic lymphoma kinase*-positive non-small cell lung carcinomas the development of secondary mutations in this gene has been described. However, the events behind crizotinib-resistance currently remain largely uncharacterized. Thus, we report on an *anaplastic lymphoma kinase*-positive non-small cell lung carcinoma patient with concomitant occurrence of *epidermal growth factor receptor* and *V-Ki-ras2 Kirsten rat sarcoma viral oncogene homolog* mutations upon development of crizotinib-resistance.

**Case presentation:**

A 61-year-old Caucasian never-smoking male was diagnosed with anaplastic lymphoma kinase -positive pulmonary adenocarcinoma, stage T4N3M1b. Treatment with crizotinib initially resulted in complete objective response in the thorax and partial response in the abdomen, but after 8 months of therapy the patient acquired resistance and progressed. Biopsies from new metastases revealed development of *epidermal growth factor receptor* and *V-Ki-ras2 Kirsten rat sarcoma viral oncogene homolog* mutations concomitant with the original *anaplastic lymphoma kinase* gene rearrangement and without signs of *anaplastic lymphoma kinase* fusion gene amplification or secondary *anaplastic lymphoma kinase* mutations.

**Conclusion:**

To our knowledge, this is the first report of an *anaplastic lymphoma kinase*-positive pulmonary adenocarcinoma, which upon emergence of crizotinib resistance acquired 2 new somatic mutations in the *epidermal growth factor receptor* and *V-Ki-ras2 Kirsten rat sarcoma viral oncogene homolog* genes, respectively, concomitant with the original *anaplastic lymphoma kinase* rearrangement. Thus, these 3 driver mutations, usually considered mutually exclusive, may coexist in advanced non-small cell lung carcinoma that becomes resistant to crizotinib, presumably because heterogeneous tumor clones utilize epidermal growth factor receptor and/or V-Ki-ras2 Kirsten rat sarcoma viral oncogene homolog signaling to circumvent the inhibition of anaplastic lymphoma kinase-mediated signaling by crizotinib. The identification of new targetable somatic mutations by tumor re-biopsy may help clarify the mechanism behind the development of the acquired crizotinib resistance and pave the way for combined strategies involving multiple targeted therapies.

## Background

Approximately 85% of newly diagnosed lung cancers are non-small-cell lung carcinoma (NSCLC). Despite surgery, radiotherapy and intensive chemotherapy, the median 5-year survival rate remains around 10% [1]. However, subsets of NSCLC harboring specific driver mutations affecting *EGFR* (*epidermal growth factor receptor*) and *ALK* (*anaplastic lymphoma kinase*) genes can obtain remarkable benefit from therapies targeting these oncogenic drivers [2]. Oncogenic *ALK* gene fusions were identified in a distinct subpopulation (approximately 5%) of NSCLC patients [3] typically characterized by adenocarcinoma histology, young age, no- or light-smoking history, and in the vast majority of cases, lack of concomitant mutations in *EGFR*- or *KRAS* (*V-Ki-ras2 Kirsten rat sarcoma viral oncogene homolog*) genes or amplification of *MET* (*mesenchymal-epithelial transition*) gene [4]. *ALK*-positive NSCLC patients have been shown to be highly responsive to the oral small-molecule triple ALK, ROS1 and MET tyrosine kinase inhibitor (TKI) crizotinib (Xalkori®; Pfizer) [5,6]. Based on phase I and II trials, showing a response rate around 60% in patients with *ALK*-rearranged NSCLC, crizotinib was approved as second-line treatment for this subset of patients by the US Food and Drug Administration in August 2011 [2] and by the European Medicine Agency in Autumn 2012. Moreover, a recent prospective randomized phase III trial has shown that crizotinib provides longer progression-free survival, higher response rates, and better quality of life than chemotherapy when used as second-line in patients with advanced, previously chemotherapy-treated, *ALK*-positive NSCLC [7]. *ALK* rearrangements typically consist of a small inversion on chromosome 2p23-21, resulting in the fusion between exon 20–29 of the *ALK* gene (encoding the kinase-domain) and exons 1–13 (different variants due to different breakpoints) in the N-terminal portion of the *Echinoderm Microtubule-associated protein-Like 4* (*EML4*) gene. Rarer *ALK* translocation partners*,* such as *Kinesin Family member 5B* (*KIF5B*) and *TRK-fused gene (TFG)* have also been reported in NSCLC. Cases of atypical translocation with partial loss of chromosomal material, resulting in so called single-red signals by fluorescence in-situ hybridization (FISH) analysis of *ALK* gene rearrangements in tumor cell nuclei, may also occur [4,8]. These different types of *ALK* rearrangements result in the expression of stabilized chimeric ALK fusion-proteins with constitutive kinase activity and oncogenic properties [3,5,9,10]. In particular, ALK fusion proteins constitutively transmit signals via PI3K/AKT/mTOR and RAS/RAF/MEK/MAPK signaling pathways, leading to enhanced cell survival and proliferation [3,11].

Although most of *ALK*-positive NSCLC patients initially show rapid and effective response to crizotinib [5,6], they tend to acquire resistance to this targeted drug, a phenomenon also seen with other targeted therapies. This is an emerging hurdle preventing ALK inhibitors from having a more prolonged beneficial effect. In some *ALK*-positive NSCLC patients the development of secondary point mutations in the *ALK* kinase domain or *ALK* fusion gene amplification have been shown to be responsible for the acquired resistance to crizotinib [9,12,13]. Although, the molecular mechanisms of acquired crizotinib resistance are beginning to emerge in some NSCLC patients, in many other patients they remain unknown [5,9,10,12,14,15]. Patients, such as the one reported here, acquiring resistance to ALK-TKI by developing mutations in different genes, could help clarify the mechanism involved in this process and the strategies to overcome it.

## Case presentation

A 61-year-old Caucasian never-smoking male was referred to hospital in June 2011 due to respiratory complains and weight loss. The initial diagnosis was pneumonia but fused imaging positron emission tomography (PET) and computerized tomography (CT) of the chest revealed an irregular tumor infiltrate of 5 × 5 cm and possible lymphangitis carcinomatosa in the left lung’s upper lobe, enlargement of bilateral mediastinal and right cervical lymph nodes, and enlargement of both adrenals glands. All these lesions displayed increased uptake of 18F-fluoro-2-deoxy-D-glucose (^18^F-FDG). Furthermore, bilateral pleural effusion and direct tumor invasion into mediastinum and pericardium was observed. All together, the TNM stage was T4N3M1b.

**Figure 1:**
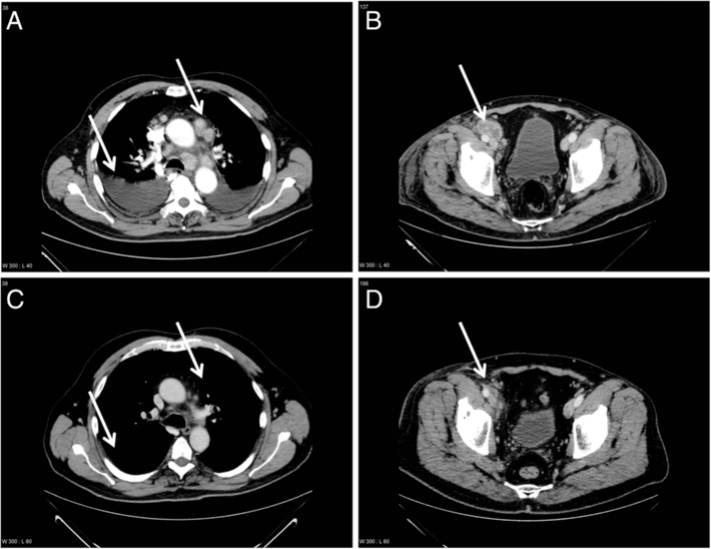
**Computerized tomography scanning of thorax (A, C) and pelvis (B, D) before initiating crizotinib treatment (A, B) and response to crizotinib (C, D). A**: Multiple malignant enlarged lymph nodes in mediastinum and bilateral pleural effusion (arrows). **B**: Malignant enlarged lymph nodes along right iliac vessels (arrow). **C**: Objective complete response in thorax without new lesions (arrows). **D**: Partial response with significant regression of metastatic lymph nodes along iliac vessels (arrow).

**Figure 2:**
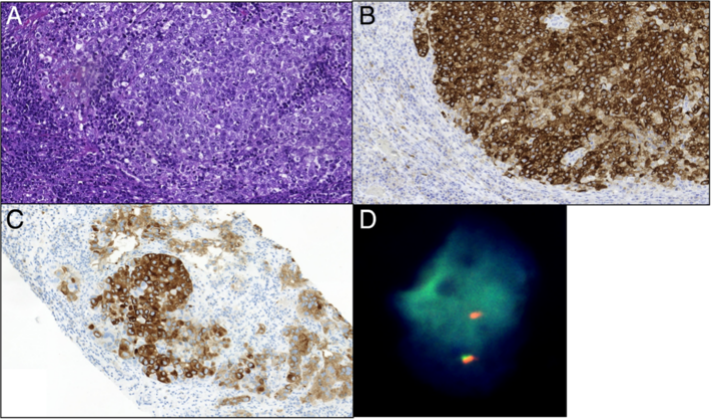
**Metastatic poorly differentiated pulmonary adenocarcinoma before (A, B) and 8 months after starting crizotinib treatment (C, D). A**: In inguinal lymph node before initiation of crizotinib treatment (HE, x20). **B**: Serial section of sample in A with strongly positive anaplastic lymphoma kinase immunostaining in the metastatic tumor tissue (5A4 mAb, x20). **C**: Anaplastic lymphoma kinase-positive immunostaining in newly appeared inguinal lymph nodal metastasis after 8 months of crizotinib treatment (5A4 mAb, x20). **D**: Fluorescence in-situ hybridization analysis of preserved anaplastic lymphoma kinase rearrangement in newly appeared inguinal lymph nodal metastasis after 8 months of crizotinib treatment. Representative tumor cell nucleus with one normal fusion signal and one single red/orange signal (Oil, x60).

A small transbronchial biopsy of the pulmonary infiltrate showed a histology and immunohistochemistry (IHC) profile (mucin stain+, CK7+, TTF1+, CK5/6-, p63-) of primary adenocarcinoma (data not shown). Because of insufficient biopsy material for molecular tests and quickly deteriorating patient performance, first-line chemotherapy including carboplatin, vinorelbine, and bevacizumab was started. Despite an initial partial response to this treatment, the disease further progressed with massive enlargement of mediastinal, retroperitoneal, and inguinal lymph nodes (Figure 1A-B). One of the right inguinal lymph nodes was excised and corresponding formalin-fixed paraffin-embedded (FFPE) sections were used to histologically and immunohistochemically confirm that the metastatic tumor tissue originated from the pulmonary adenocarcinoma (same morphology of mucin-producing adenocarcinoma and IHC-profile with CK7+, TTF1+, CK20-, CDX2-, PSA-). Further sections from the metastasis were utilized to perform *EGFR*- and *KRAS*-mutation analysis. Nested PCR-amplification followed by direct sequencing of exon 18–21 of the *EGFR* gene and by pyrosequencing of codon 12, 13, 59 and 61 of the *KRAS* gene was performed. No *EGFR* or *KRAS* mutations were identified. However, IHC with monoclonal antibody against the *ALK* gene product (Novocastra, Clone 5A4), revealed intense positive staining in the metastatic tumor tissue (corresponding to 3+ according to the algorithm proposed by Thunnissen E. et al. [8]) (Figure 2A-B). FISH analysis of the specimen with dual-color break-apart rearrangement probe (Vysis LSI ALK; Abbott Molecular), detected *ALK* rearrangement in 40% of the analyzed tumor cell nuclei (100 tumor cell nuclei analyzed with a cut-off of 15%). The cell nuclei with rearrangement appeared with one normal fusion signal and an abnormal single red signal for *ALK*, characteristic for *ALK* gene fusion with partial deletion of genetic material [4,8]. The results provided indication for treating the patient with the ALK inhibitor crizotinib.

Meanwhile, the patient had been hospitalized because of sudden deterioration of general condition and dyspnea caused by malignant bilateral pleural effusion and hemodynamically significant, cytologically confirmed, pericardial effusion requiring echocardiography-guided pericardiocentesis with 1700 ml fluid decompression. After improvement of his general condition the patient started second line treatment with crizotinib. This drug was provided to the patient through compassionate use program, as it was not approved in Denmark at that time. The patient responded positively to crizotinib with complete remission in the thorax and partial remission in the abdomen and pelvis (Figure 1C-D).

**Figure 3:**
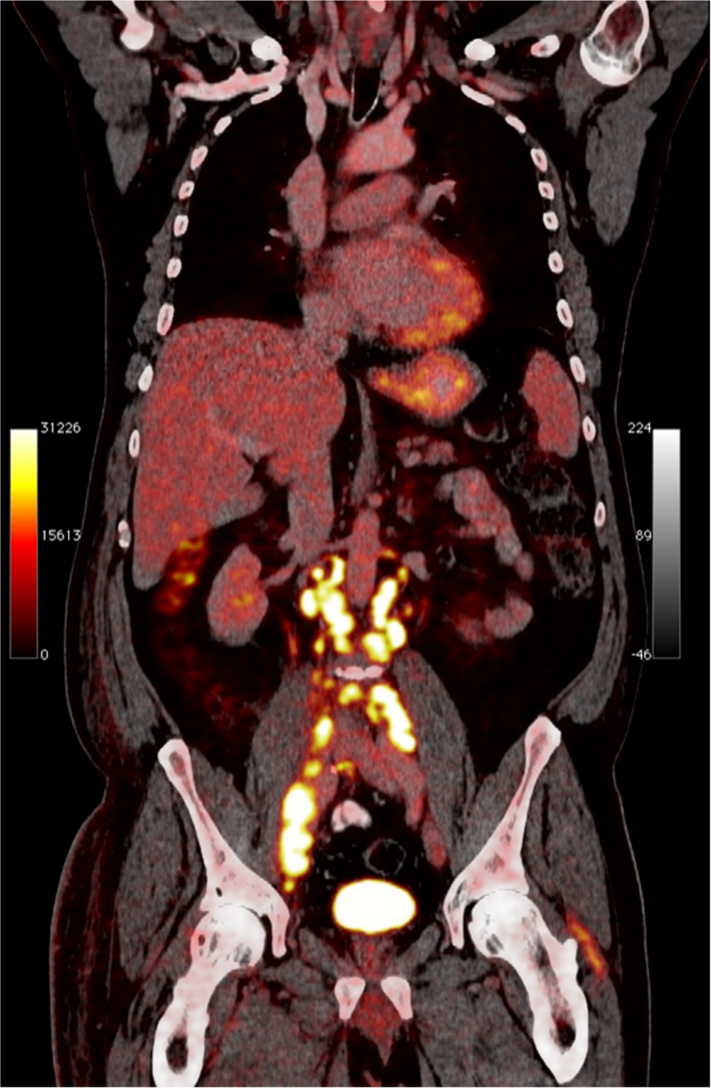
Positron emission tomography scanning after 8 months of crizotinib treatment: still complete response in the thorax but marked disease progression in the abdomen with newly appeared metastases in retroperitoneal, pelvic and inguinal lymph nodes.

**Figure 4:**
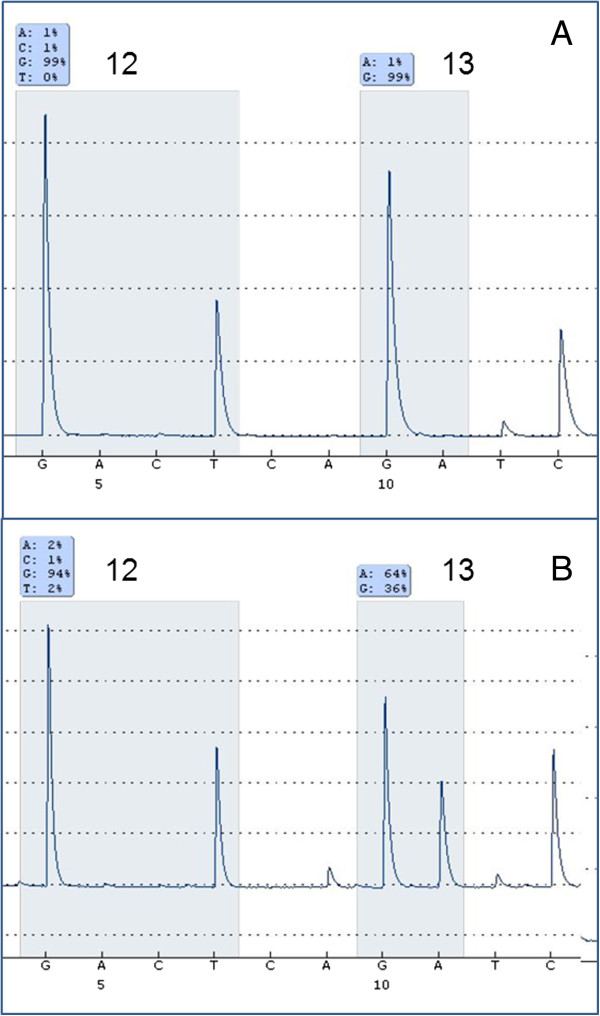
***Ki-ras2 Kirsten rat sarcoma viral oncogene homolog *mutation analysis by pyrosequencing. A**: *V-Ki-ras2 Kirsten rat sarcoma viral oncogene homolog* wild type codon 12 + 13 in inguinal lymph node metastasis before starting crizotinib treatment **B**: *V-Ki-ras2 Kirsten rat sarcoma viral oncogene homolog* mutation (G13D; GGC > GAC) in newly appeared inguinal lymph nodal metastasis after 8 months of crizotinib treatment.

**Figure 5:**
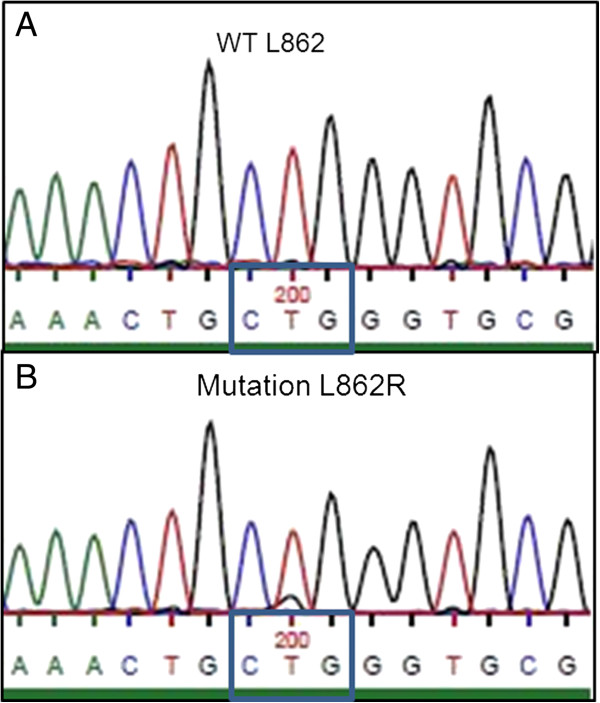
***Epidermal growth factor receptor *mutation analysis by Sanger sequencing. A**: *Epidermal growth factor receptor* wild type codon 862 (L862) in inguinal lymph node metastasis before starting crizotinib treatment **B**: *Epidermal growth factor receptor* mutation in codon 862 (c.2585 T > G; p.L862R) in newly appeared inguinal lymph nodal metastasis after 8 months of crizotinib treatment.

However, after 8 months of treatment the patient displayed massive disease progression in the retroperitoneal, pelvic and inguinal lymph nodes, while there was still complete response in the thorax (Figure 3). In order to investigate the discrepant response in the thorax and the retroperitoneum/pelvis and the underlying mechanisms of crizotinib resistance, a new needle biopsy from the relapsed tumor tissue in the inguinal lymph nodes was obtained. The histology and IHC-profile were the same as in the primary lung adenocarcinoma and in the previous biopsy from inguinal metastasis (same histological appearance, mucin stain+, CK7+, TTF1+, CK20-, CDX2-, PSA-; data not shown). Moreover, clinico-radiological analyses showed no tumors in the gastrointestinal- or genito-urinary tract. All together the data, despite the relatively unusual metastatic pattern for a NSCLC, excluded the possibility of spreading from a second primary adenocarcinoma located in an abdominal/pelvic organ. FISH and IHC revealed that the original *ALK* rearrangement was still present, as we detected 60% of examined tumor cells with single-red *ALK* signal by FISH and intensely positive (3+) IHC-staining for ALK protein in the tumor tissue (Figure 2C-2D). Importantly, we did not detect amplification of the rearranged *ALK* gene nor secondary point mutations in the kinase-domain-coding exons 21–25 of *ALK* gene, which previously have been reported as possible mechanisms of acquired crizotinib resistance [10,12,13]. However, mutations in codon 13 of *KRAS* gene (c.38G > A, p.G13D) (Figure 4) and in exon 21 of *EGFR* gene (c.2585 T > G, p.L862R) were detected in the relapsed metastasis (Figure 5). Additional mutation analysis of genomic DNA re-isolated from the metastatic inguinal tumor tissue initially examined before the start of crizotinib treatment, confirmed that this was without *EGFR* or *KRAS* mutations. The results could indicate that the mutations detected in the relapsed tumor tissue were newly occurred; however these mutations may have also emerged as a result of selective pressure induced by crizotinib and 1 or more of the previous therapies. It is indeed possible that these mutations were already present at baseline in clones of the primary lung tumor representing so small a fraction of the original tumor population to go undetected by our sequencing techniques. Similarly, the co-existence of *KRAS* and *EGFR* mutations in the same tumor tissue could be interpreted as either double mutation in the crizotinib-resistant tumor cells or more likely as the occurrence of two different tumor subpopulations with constitutive activation of either the EGFR or the KRAS signaling pathway as different mechanisms of acquired crizotinib-resistance. Neither of these 2 scenarios could be excluded by the methods used in our molecular analysis. The *EGFR* mutation L862R has to our knowledge not previously been described in the literature, but given its proximity to mutations known to be sensitive to EGFR-TKI (L858R and L861X), tumor clones bearing the L862R mutation could also be potentially responsive to EGFR-TKI. Meanwhile, because of rapid worsening while waiting for these new molecular tests the patient received third-line pemetrexed, showing further progression in abdomen and pelvis, but still no sign of relapse in the thorax. Unfortunately, the patient rapidly deteriorated and died before being able to attempt a palliative radiation of the lymph nodes in pelvis and a fourth-line treatment with erlotinib.

## Discussion

Multiple molecular mechanisms are emerging as causes of acquired resistance to treatment with TKI like crizotinib, posing new challenges for targeted therapy of NSCLC. We have presented a patient with advanced, *ALK-positive, EGFR and KRAS* wild-type, pulmonary adenocarcinoma, who after 8 months of response to crizotinib treatment acquired resistance to this drug and progressed rapidly. Re-biopsy of relapsed metastatic tumor tissue showed newly emerged somatic mutations in both the *KRAS* and the *EGFR* gene, while the tumor cells still carried the original *ALK* rearrangement but without signs of *ALK* fusion gene amplification or secondary *ALK* mutations. These results indicated that the observed tumor progression was not due to one of the known mechanisms of acquired crizotinib resistance, *i.e.* loss of *ALK* rearrangement, amplification of *ALK* fusion gene, or secondary *ALK* mutations [9,13,15], but was most likely due to the emergence of concurrent *EGFR* and *KRAS* mutations. To our knowledge, this is the first report of a concomitant presence of *ALK* gene rearrangement, *EGFR* mutation, and *KRAS* mutation in the same NSCLC patient. Although the vast majority of *ALK*-positive pulmonary adenocarcinomas lack concomitant *EGFR*- or *KRAS*-mutations, suggesting that these mutations are mutually exclusive [4,12,16], single cases with concurrent *EML4-ALK* fusion gene and *EGFR*-mutations have been reported [17-20]. These single patients reportedly received EGFR-TKI therapy with variable effect, but none of these patients were treated with crizotinib. In contrast, the initial biopsy from the metastatic tumor in the current case showed only *ALK* rearrangement and wild-type *EGFR* and *KRAS*, while re-biopsy from a new metastasis at progression after 8 months of crizotinib treatment revealed the appearance of *EGFR* and *KRAS* mutations in addition to an unchanged *ALK* rearrangement. This suggests that these 3 mutations, typically considered mutually exclusive [4,12,16], can coexist in some cases of NSCLC that become resistant to crizotinib *in vivo,* presumably because the tumor cells or heterogeneous tumor clones utilize EGFR and/or KRAS signaling to circumvent the inhibition of ALK-mediated signaling by crizotinib. The emergence of either *EGFR* or *KRAS* mutations have been reported in single NSCLC cases acquiring resistance to crizotinib, both with and without concomitant loss of *ALK* gene rearrangement [15].

Metastatic spreading of NSCLC to subdiaphragmatic lymph nodes, as observed in our case, is relatively uncommon compared to other metastatic sites, such as the liver. Indeed, a recent study of 1,191 NSCLC patients consecutively staged by ^18^F-FDG PET/CT scan, showed that 6% of them had metastasis to abdominal lymph nodes and only 0.5% to external iliac lymph nodes [21]. However, whether the rare occurrence of three simultaneous oncogenic mutations had an impact on this unusual metastatic pattern in our patient is unknown.

Together with previous reports, our case clearly underlines the need for re-biopsy in patients who develop acquired resistance to a TKI such as crizotinib. This should be done primarily to investigate the mechanisms behind the acquired resistance, and hopefully identify new somatic mutations that potentially could be targeted. The appearance of new targetable somatic mutations could pave the way for combinatorial therapeutic strategies involving multiple targeted therapies. In this case, however, the presence of the G13D *KRAS* mutation could render the patient resistant to an EGFR-targeting therapy. This could however not be verified since the patient died before a new targeted treatment could be initiated. Nevertheless, the possibility of treating the patient with the EGFR-TKI erlotinib was considered. Indeed, even if the co-existence of *KRAS* and *EGFR* mutations could be interpreted as double mutation occurring in the crizotinib-resistant tumor cells, the possibility of different tumor clones with activation of EGFR and KRAS signaling as different mechanisms of crizotinib-resistance appeared to be an alternative, perhaps more likely explanation. Furthermore, the identified L862R *EGFR* mutation, although not reported before, was located near mutations known to be sensitive to EGFR-TKI, such as L858R and L861X. Therefore, the tumor clone bearing this *EGFR* mutation could have been potentially responsive to EGR-TKI therapy. However, a previously described single case of L862V *EGFR* mutation was associated with lack of response to gefitinib and the same could have been the scenario with the L862R *EGFR* mutation. Functional characterization of this and other rare *EGFR* mutations is needed in order to elucidate their impact on treatment with TKI [22].

The appearance of new *EGFR* and *KRAS* mutations could indicate a shift in the oncogenic survival and proliferation dependency from the ALK signaling pathway to EGFR and/or KRAS pathways in the ALK-TKI resistant cells. Activation of HER-family proteins has been associated with sustained down-stream signaling in the presence of ALK-TKI, indicative of the shift in survival dependency from the ALK signaling pathway to HER-family-driven pathways in the ALK-TKI resistant cells [14]. Cell line experiments have also shown that both EGFR and MET activation and the interaction between the corresponding signaling pathways may mediate crizotinib resistance [9], suggesting that both signaling by EGFR and MET may be crucial for the survival of lung cancer cells upon ALK inhibition. *KIT* amplification, auto-phosphorylation of EGFR, and aberrant expression of other receptor tyrosine kinases have also been postulated by *in vitro* experiments to cause resistance to crizotinib in *ALK*-rearranged cancer cells [9,13,15].

However, the mechanisms behind crizotinib resistance in NSCLC patients are still poorly understood. NSCLC is increasingly recognized as heterogeneous set of diseases at the morphological and molecular level and these differences may drive therapeutic decision making. Tumor heterogeneity, the invariable development of resistance to targeted monotherapy with TKI, and the occurrence of multiple driver mutations in TKI-resistant tumor cells, appear to be problems strictly related to one another and capable of undermining effective treatment. Whether separate oncogenic driver subclones with *EGFR* and *KRAS* mutations can arise completely independently, they share a common progenitor that acquires these drivers independently as later events, or both drivers coexist within the same cell, and then one or other is lost in subclonal evolution is still unclear. Regardless of which evolutionary clonal process takes place, the potential role for combinatorial therapeutics in overcoming crizotinib resistance in the clinic appears of increasing importance [9,15].

## Conclusion

Our case demonstrates the utility of tumor re-biopsies in order to better understand the mechanisms of crizotinib resistance in patients with *ALK*-rearranged pulmonary adenocarcinoma. Moreover, our case indicates that *ALK*-rearranged NSCLC may possibly acquire resistance to crizotinib through the emergence of concurrent somatic mutations in both *EGFR* and *KRAS* genes. The concomitant occurrence of *EGFR* and *KRAS* mutations with *ALK* rearrangement upon crizotinib resistance suggests that the most effective therapeutic strategy for ALK-positive lung cancers may ultimately require combined strategies targeting not only *ALK* gene fusions and *ALK* resistance mutations, but also other driver mutations that activate alternative TKI-resistance pathways.

## Consent

Written informed consent was obtained from the wife of the deceased patient for publication of this Case report and any accompanying images. A copy of the written consent is available for review by the Editor of this journal.

## Competing interests

Eric Santoni-Rugiu has received speaker fees from Pfizer and Roche as well as research grants from Pfizer. Jens Benn Sørensen has received speaker fees and research grants from Pfizer.

Edyta Maria Urbanska has received speaker fees from Pfizer.

The other authors declare that they have no competing interests.
